# Translational implications of circadian activity alterations in an experimental model of late-onset depression induced by prenatal excess of glucocorticoids

**DOI:** 10.3389/fnbeh.2025.1620800

**Published:** 2025-09-10

**Authors:** Stefan Spulber, Raj Bose, Frederik Elberling, Mirko Conti, Sandra Ceccatelli

**Affiliations:** Department of Neuroscience, Karolinska Institutet, Stockholm, Sweden

**Keywords:** depression, glucocorticoids, fetal origin of adult disease, circadian activity pattern, neurogenesis

## Abstract

Most neuropsychiatric conditions, including neurodevelopmental disorders, can have different etiology depending on genetic influences, environmental factors, and gene-environment interactions. Consistent evidence points to low birth weight, commonly associated with prenatal exposure to excess glucocorticoids (GC), as risk factor for neuropsychiatric disorders including depression, ADHD and schizophrenia. In this review we give an overview of our behavioral and mechanistic studies linking prenatal exposure to GC to depression. The behavioral analyses in our mouse model revealed that prenatal exposure to synthetic GC dexamethasone (DEX) alters hippocampal neurogenesis and induces depression-like behavior that responds differently to antidepressive therapies. Using neural progenitor cells as an *in vitro* experimental model, we could show changes in the methylation state of genes regulating proliferation, differentiation, and migration suggesting that epigenetic modifications are involved in neurogenesis alterations induced by GC. A particularly interesting observation was the alteration in circadian patterns of activity accompanied by weaker coupling between the central clock and peripheral oscillators preceding the late onset of depression in mice exposed to DEX *in utero*. The results suggest that alterations in patterns of circadian spontaneous activity may predict the onset of depression and the response to therapy in depressed patients. Our collaborative clinical investigations provide evidence for the prognostic value of circadian activity analysis in predicting the response to antidepressant treatments in patients affected by major depressive disorder.

## Etiology of depression

Major depressive disorder (MDD) is a common neuropsychiatric disorder, often chronic or recurrent, with a significant negative impact on the overall health, social, and professional functioning of affected individuals. The variety of symptoms across depressed persons includes emotional, cognitive and behavioral aspects making depression a multifaced disorder. According to the Diagnostic and Statistical Manual of Mental Disorders (DSM-5), the requirement for 5 or more criteria to be fulfilled yields almost 1500 possible combinations leading to a diagnosis of MDD (Østergaard et al., 2011). The etiology of MDD is complex and includes genetic and environmental components. In contrast with other psychiatric disorders (e.g., schizophrenia or bipolar disorder, where genetic contribution accounts for 60%–90% of the cases), heritability has been estimated between 30 and 40% (Franklin et al., 2025). A recent meta-analysis identified 102 genetic variants associated with MDD (Howard et al., 2019) and highlighted potential links with neurodevelopmental disorders such as attention deficit hyperactivity disorder (ADHD) and schizophrenia, via shared risk genes. The impact of environmental factors, including childhood adversity and other stressful conditions, have been shown to be mediated by epigenetic changes (Yuan et al., 2023) in genes related to the hypothalamic-pituitary-adrenal (HPA) axis, also relevant for depression and anxiety (Alahmad et al., 2025).

### Developmental origins of depression

The hypothesis of developmental origins of health and disease (DOHaD) posits that an adverse intrauterine environment alters the developmental trajectory, resulting in structural and functional changes in target tissues/organs (Cao-Lei et al., 2020). The concept is based on Barker’s early investigations indicating that cardiovascular diseases in adult life may have their origin during development (Barker, 1994). Factors affecting the maternal wellbeing throughout gestation (i.e., placenta dysfunctions, stress, malnutrition, metabolic syndrome, infections, and exposure to toxic insults) may have a negative impact on fetal growth and neurodevelopment with long-term consequences. Intrauterine growth restriction (IUGR) has been defined as abnormal adaptation of fetal development to an adverse prenatal environment (Armengaud et al., 2021), and mounting evidence link IUGR to higher risk of psychiatric disorders (Räikkönen et al., 2008; Strang-Karlsson et al., 2008; Anacker et al., 2014). An adverse perinatal environment appears to have detrimental effects not only on HPA axis regulation, but also on the programming of the suprachiasmatic nucleus (SCN), leading to alterations in circadian rhythms often associated with depression in humans [reviewed in Kennaway (2002)].

### The HPA-axis and glucocorticoid signaling

Glucocorticoids (GC) are a class of steroid hormones secreted by the adrenal glands that have a critical role in mediating the stress response. GC are released in pulses of varying amplitude, with several peaks and troughs within a 24-h cycle. They support the organogenesis of the central nervous system by initiating terminal maturation of neural progenitors, remodeling of axons and dendrites, and promoting cell survival (Meyer, 1983; Yehuda et al., 1989; Cameron and Gould, 1994). In the human fetus, endogenous secretion of GC has a first peak between 7 and 14 weeks postcoitum; continues at very low levels before it begins to increase by the end of the second trimester; and a surge in serum cortisol can be observed during the last weeks before birth [reviewed in Busada and Cidlowski (2017)]. Essential for immediate postnatal survival, GC promote lung maturation and initiates surfactant production (Ward, 1984; Harris and Seckl, 2011; Khulan and Drake, 2012), which led to the use of synthetic GC administration to expecting women at risk of preterm delivery in order to reduce the risk of infant mortality (Liggins and Howie, 1972). Despite this short-term benefit of exogenous GC agonists, epidemiological studies have revealed long-term risks of chronically altered HPA-axis response to stress (Waffarn and Davis, 2012). Animal models of prenatal exposure to excess GC have shown decreased GR in the hippocampus (Levitt et al., 1996) which results in HPA-axis hypo-responsiveness (Sloboda et al., 2007), and increased susceptibility of neuronal cells to oxidative stress associated with altered antioxidant defenses (Ahlbom et al., 2000). The long-term effects of exposure to excess GC depend on the timing of exposure and involve epigenetic modifications which can be passed to the offspring (Drake et al., 2005, 2011).

Experimental data indicate a bidirectional connection between depression on one side, and stress and GC secretion on the other side. Chronic stress leads to depression in animal models (Antoniuk et al., 2019) and humans (Ding and Dai, 2019) alike. In addition, both baseline GC secretion and the stress response are altered in depression patients (Powers et al., 2016) with distinct sex-differences in HPA-axis reactivity and stress generation (Slavich and Sacher, 2019). Adverse perinatal events have long-lasting effects on HPA programming and function at adult age. Experimental and epidemiological studies have shown that developmental exposure to excess GC alters the function of the HPA-axis and increases the risk for mental disorders, including depression (Cottrell and Seckl, 2009; Moisiadis and Matthews, 2014; Spulber et al., 2015; Laugesen et al., 2025). Recent studies report higher risk for mental health disorders in children exposed to GC during pregnancy (Räikkönen et al., 2020), with adjusted relative risk for mood, anxiety and stress-related disorders of 1.5 (Laugesen et al., 2025). In populations where data from longer follow-up times were available, epidemiological studies show that IUGR increases the risk of developing depression in adulthood (Räikkönen et al., 2008; Strang-Karlsson et al., 2008; Pesonen et al., 2009; Grissom and Reyes, 2013; Longo et al., 2013).

### Depression and circadian rhythms

**FIGURE 1:**
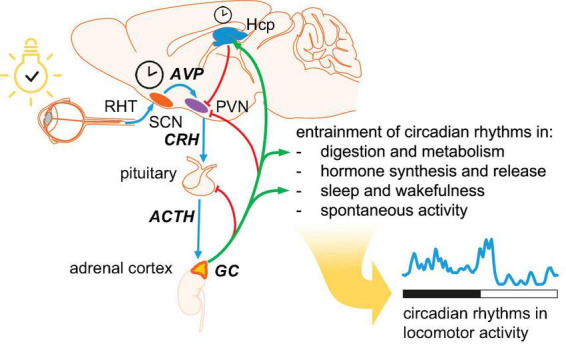
Hierarchical organization of circadian oscillators. The central clock is located in the suprachiasmatic nucleus (SCN). Photic entrainment (blue arrows) starts with information about environmental light from retinal ganglion cells conveyed to the SCN via the retinohypothalamic tract (RHT). Arginin-vasopressin (AVP) from SCN shell area is released in the hypothalamic paraventricular nucleus (PVN) to regulate the secretion of corticotropin-releasing hormone (CRH). CRH triggers the release of adrenocorticotropic hormone (ACTH), which drives the pulsatile secretion of glucocorticoids (GC) from adrenal cortex. Circulating GC provide negative feedback at several levels in the HPA axis (red arrows) and entrain peripheral oscillators (green arrows). Circulating GC reaching the hippocampus (Hcp) maintain the pool of neural stem cells and drive hippocampal neurogenesis. In addition, the Hcp provides negative feedback to PVN.

Regulation of homeostasis in anticipation of relevant changes in environment is of paramount importance for any organism, not only for adjusting the biological rhythms to the time of day, but also for predicting the coming changes such as approaching the transition between light and dark period. The core molecular clock consists of transcription-translation feedback loops (TTFL), that is specific transcription factors encoded by clock genes. While most mammalian cells express functional molecular clocks, only the neurons in the suprachiasmatic nucleus (SCN) – the central clock, located in the anterior hypothalamus - possess mechanisms to synchronize oscillations at population level (Ko and Takahashi, 2006). Outside the SCN, external signals from a master clock are required for synchronizing the molecular clocks across organs and systems, and this is achieved primarily by GC signaling (Balsalobre et al., 2000; Son et al., 2011; Albrecht, 2012; Figure 1). Circadian rhythms in the HPA axis are entrained by arginine-vasopressin (AVP) released from the SCN into the paraventricular hypothalamic nucleus (PVN) to regulate the release of corticotropin-releasing hormone (CRH) (Kalsbeek et al., 2010, 2012).

Depression is associated with disruption of sleep and alterations of circadian rhythms (Vadnie and McClung, 2017; Lyall et al., 2018; Mendoza, 2019). Notably, insomnia and hypersomnia are both listed among diagnostic criteria for MDD (American Psychiatric Association [APA], 2013) and either symptom have a significant impact on patients’ quality of life. Several theories aiming to explain the occurrence of sleep problems in MDD patients have been developed based on the two-process model of sleep regulation (Borbely, 1982) and the neurotransmitter imbalance hypothesis [reviewed in Wang et al. (2015), Riemann et al. (2025)]. The association between depression and circadian dysregulation is supported by several lines of evidence. First, genetic association studies have identified association between depression and clock gene variants for core clock genes (Lavebratt et al., 2010; Sjoholm et al., 2010; Gyorik et al., 2021). Similarly, seasonal affective disorder has been associated with combinations in core clock gene variants (Partonen et al., 2007). Post-mortem studies have identified disruption of clock genes expression in the brains of people suffering from depression (Sequeira et al., 2007, 2012), and the severity of depression symptoms is associated with the degree of misalignment of circadian rhythms (Courtet and Olié, 2012). In rodents, Bmal1 knock-down in the SCN (Landgraf et al., 2016), or manipulation of the light-dark cycle (Ben-Hamo et al., 2016) can result in depression-like behavior. Second, circadian disruption by shiftwork was associated with an increased risk to develop depression (Logan and McClung, 2019). A recent large population study indicates that blunted circadian rhythms of activity are associated with an increased lifetime risk for depression and mood instability (however not satisfying the diagnostic criteria for unipolar or bipolar depression) (Lyall et al., 2018). Lastly, several antidepressants produce changes in circadian features and some therapeutic approaches involving chronotherapy and wake and light therapy have proven effective in certain cases (Wang et al., 2015; Wichniak et al., 2017; Humpston et al., 2020; Silva et al., 2021). Antidepressant pharmacotherapy includes drugs targeting serotonin (5-HT), dopamine (DA), and norepinephrine (NE) signaling, and have a direct impact on circadian rhythms [reviewed in Lee et al. (2022), Sato et al. (2022)]. This can be explained by the input to the SCN from various neurotransmitter systems. Thus, serotoninergic input from the median raphe tonically inhibits the glutamate release from the retinohypothalamic tract (RHT) via activation of both presynaptic and postsynaptic receptors (Selim et al., 1993; Quintero and McMahon, 1999; Sanggaard et al., 2003), thereby weakening photic entrainment of the SCN. In addition, 5-HT can shift the phase of neuronal activity in the SCN by regulating clock gene expression (Horikawa et al., 2000). Dopaminergic input from the ventral tegmental area (VTA) facilitates re-entrainment of circadian rhythms of activity via tonic activation of D1 (Drd1) receptors (Grippo et al., 2017; Grippo and Güler, 2019). DA and 5-HT are also the main neurotransmitters involved in non-photic entrainment of circadian rhythms (e.g., entrainment by physical activity or restricted feeding or by social interactions). Noradrenergic input to the SCN has been suggested early on (Cagampang et al., 1994), and noradrenaline reuptake inhibitors shift the phase of the circadian clock (Vacher et al., 2003; O’Keeffe et al., 2012). Suppressing the molecular clock function by enhancing the negative arm of the TTFL in the prefrontal cortex of mice not only induced depression-like behavior, but also mitigated the antidepressant effects of ketamine (Sarrazin et al., 2024).

## Modeling depression in experimental animals

Given the heterogeneity of depression presentation (reliance on subjective reporting of feelings and mood), it is not surprising that the development of experimental models of depression and relevant behavioral tests has been a challenging task. In addition, the complex interplay between genetics, environmental and psychosocial aspects exclude the possibility to envisage a model that replicates what is observed in patients. However, proxy measures for the core symptoms have been developed in rodents, namely learned anhedonia and learned helplessness. Anhedonia is evaluated by measuring the bias toward consumption of sweetened vs. regular water (sucrose preference). These tests assess essentially the response to reward, depend heavily on the experimental setting, and carry limited information out-of-context (Sahin, 2023). Learned helplessness is assessed by exposing the animal to an unescapable aversive situation, such as suspension by the tail (tail suspension test, TST), or immersion in a water-filled cylinder (forced swimming test, FST), then measuring the total time spent not trying to escape (immobility time). The tests, originally developed in the 1970’s and 1980’s (Porsolt et al., 1977; Steru et al., 1985) have been extensively used for drug discovery, but their validity for assessing depression in animal models remains debatable (Borsini and Meli, 1988). Recent studies have shown differences between acute and chronic treatment for the same feature, as well as differences between drugs acting depending on the neurotransmitter signaling involved (Holmes et al., 2002; Cryan et al., 2005). It is therefore recommendable to be interpreted as “depression-like behavior” or “depression-related behavior” (Sahin, 2023).

**FIGURE 2:**
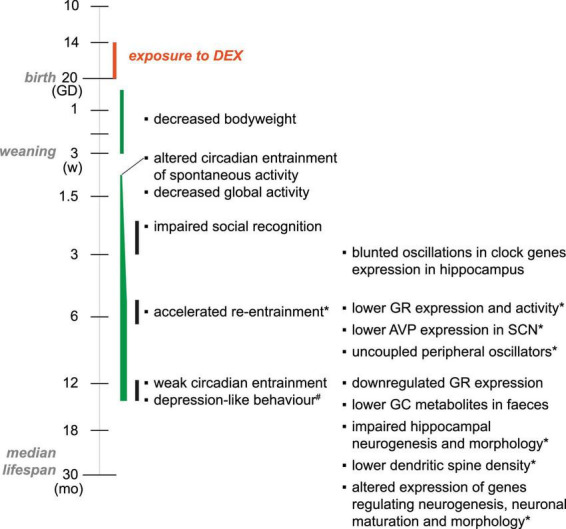
Timeline of behavioral alterations and associated mechanisms leading to onset of depression-like behavior in male mice exposed to DEX *in utero*. The axis of age is logarithmic, and starts at gestational day (GD) 10, when the cortical sublate is defined and cortical neurogenesis begins. Endogenous secretion of GC starts at GD14. IUGR induced by exposure to DEX *in utero* is confirmed by the decrease in bodyweight over the first 3 weeks (w) after birth. The core alterations associated with the depression-like behavior (i.e., altered circadian entrainment and decreased GR-signaling) are observed by 5–6 months (mo) of age. The mechanisms investigated are listed in the right column. *Alterations reversed by DMI treatment at respective age. #Depression-like behavior was not reversed by Fluoxetine (SSRI class antidepressant), but DMI (SNRI class antidepressant) reduced immobility time in FST. Remarkably, DMI treatment at 6 mo also prevented the onset of depression-like behavior at 12 mo.

Experimental models of depression build on validated risk factors identified in patients and are typically based on manipulating (1) the environment (e.g., developmental insults, or exposure to chronic stress at adult ages); or (2) biological underpinnings, at gene expression (e.g., mutations, deletions, or overexpression), or neuronal circuit level (e.g., optogenetic control of specific neuronal populations, or targeted lesions) (Planchez et al., 2019). In experimental models where depression-like behavior is induced in adult animals, such as chronic stress or social defeat, the validity of behavioral endpoints has been questioned as to whether they are physiological (i.e., adaptive) or pathological, particularly because they are most often transient (Krishnan and Nestler, 2011). In contrast, experimental models based on perinatal adversity induce persistent behavioral alterations associated with reprograming of HPA axis function which leads to maladaptive response to stress. This provides “biologically plausible” support for causal relationship and mechanistic investigations (Fitzgerald et al., 2021). Our research has been focusing on the developmental origin of neuropsychiatric disorders with special focus on the impact of prenatal exposure to excess GC. Prenatal stress or exposure to exogenous GC has been shown to lead to low birth weight in rodents [see meta-analysis in Burgueño et al. (2020)]. In our model, timed-pregnant C57Bl/6 dams were injected daily with 0.05 mg/kg/day dexamethasone (DEX, a synthetic GC analog) from gestational day (GD) 14 until delivery (Figure 2). This dose was chosen to induce moderate fetal growth retardation without affecting litter size, gestational length or maternal behavior (Spulber et al., 2015; Conti et al., 2017). Endogenous synthesis of GC in mice starts around GD14 (Michelsohn and Anderson, 1992) and feedback control of HPA axis is detected around GD16 (Reichardt and Schütz, 1996), which means that the exposure window covers the embryonic development of the HPA axis. During the first weeks after birth, the phenotype of DEX-exposed offspring was rather mild, without significant differences between males and females. A decrease in bodyweight of about 5% was present from birth until 3 weeks of age (Spulber et al., 2015), and increased frequency of ultrasonic vocalizations (USV) on PND12 (unpublished observations). Sex-related differences emerged during adolescence, when only male exposed offspring displayed increased spontaneous exploration and impaired social recognition. At adult stages, DEX-exposed males display progressively weaker circadian entrainment of spontaneous activity and develop depression-like behavior around 12 months (mo) of age. In contrast, DEX-exposed females display stronger circadian entrainment of spontaneous activity and are spontaneously hyperactive as compared to controls.

### Heritable effects in neuronal progenitors

The timeline of development of behavioral alterations following *in utero* exposure raises the question whether the effects of DEX on neuronal progenitors are persistent. Low-level DEX exposure (1microM) decreases the proliferation rate without altering survival or differentiation of embryonic neural stem cells and was associated with upregulation of senescence-related markers, such as cell-cycle regulators p16 and p21. The alterations were long-lasting, and were detectable also in daughter cells, i.e., cells which were not directly exposed to DEX (Bose et al., 2010). Persistently increased sensitivity to oxidative stress was also observed in daughter cells (Raciti et al., 2016). The persistent phenotype suggested epigenetic alterations. Indeed, we found global DNA demethylation associated with downregulation of DNA-methyl transferases (Dmnts) responsible for both maintenance of DNA methylation patterns across mitosis cycles (Dnmt1), and *de novo* methylation (Dnmt3a, Dnmt3b) (Bose et al., 2010). In addition, the upregulation of ten-eleven translocation oxygenase 3 (Tet3), an enzyme initiating the chain of reactions leading to removal of methyl groups from cytosine, mediates the epigenetic effects of GC exposure in embryonic neuronal stem cells (NSCs) (Bose et al., 2015). We found similar alterations in gene expression regulation in tissue samples collected from pups exposed to DEX *in utero*. Briefly, the expression of Dnmt3a was downregulated, Tet3 was upregulated, and global DNA methylation was decreased in PND3 pups as compared to controls (Bose et al., 2015). An in-depth analysis of differentially methylated regions (DMRs) identified altered methylation in the promoter region of genes relevant for the phenotype, including Dkk1, which mediates the acute effects of DEX in neuronal progenitors (Moors et al., 2012), and Txnip and Cyba, which are relevant for the increased susceptibility to oxidative stress (Bose et al., 2010). Notably, Txnip and Cyba expression was upregulated also in the cortex of PND3 pups exposed to DEX *in utero* (Bose et al., 2015). Taken together, these data indicate that exposure to GC during early developmental stages has persistent effects on neural stem cells, which are mediated by epigenetic changes.

### Neuroplasticity and hippocampal neurogenesis

Neuroimaging studies found consistent hippocampal atrophy in patients with depression. In addition, experimental models and post-mortem investigations have shown significant synaptic atrophy, as well as decreased hippocampal neurogenesis and altered dendritic arborization, which are consistent with cognitive impairment associated with chronic depression (Rock et al., 2014). It is worth noting that hippocampal atrophy is also found in patients experiencing the first episode of depression (i.e., it is not limited to chronic or recurrent cases) (Cole et al., 2011), which is consistent with a neurodevelopmental origin of depression. The functional outcome of altered neuroplasticity has been suggested to be a negativity bias accompanied by cognitive and emotional inflexibility (Page et al., 2024). Down-regulation of neurotrophins, such as brain-derived neurotrophic factor (BDNF) and nerve growth factor (NGF), has been suggested to play a role in decreased neurogenesis and onset of depression-like behavior.

Hippocampal neurogenesis was impaired in DEX-exposed male mice (Spulber et al., 2015; Conti et al., 2017). More specifically, both proliferation of neuronal progenitor, and neuronal differentiation were reduced as compared to controls. We performed a detailed analysis of morphology of newborn neurons expressing green fluorescent protein (GFP) delivered by retroviral infection (Conti et al., 2017). The morphological alterations in DEX-exposed males consisted mainly of reduced complexity of dendritic arborization and decreased density of dendritic spines. In addition, we observed a conspicuous increase in frequency of a particular neuronal morphology characterized by very early branching of the main dendrite, which gives a V-shaped aspect instead of the most common Y-shape morphology. The V-shaped morphology of granule neurons in the dentate gyrus has previously described to be associated with neuroinflammation (Einstein et al., 1994; Lee et al., 2015; Llorens-Martín et al., 2016), and may have profound functional consequences (Fitzsimons et al., 2013). The alterations in neurogenesis and neuronal morphology were corroborated by alterations in mRNA expression for cell cycle inhibitors (upregulated p16 and Cdkn1c) and proteins regulating neuronal differentiation and the maturation of granule cells (downregulated TrkB, GAP-43, DISC1, and Reln). Hippocampal neurogenesis is dependent on pulsations in GC secretion (Fitzsimons et al., 2016; Schouten et al., 2020; Eachus and Ryu, 2024). DEX-exposed mice display lower levels of GC metabolites in feces, and smaller diurnal variations as compared to controls (Spulber et al., 2015), which suggests dampened circadian oscillations in GC secretion. The investigation of GR expression in the hippocampus also showed significant downregulation (Spulber et al., 2015). This suggests an overall decrease in GC signaling which could explain the alteration in hippocampal neurogenesis. In experimental models, effective selective serotonin reuptake inhibitors (SSRI) antidepressants restore hippocampal neurogenesis, and blocking hippocampal neurogenesis prevents the antidepressant effects (Duman et al., 2001; David et al., 2009; Nollet et al., 2012). Notably, antidepressant effects have been suggested to be linked to effective restoration of hippocampal neurogenesis (Fitzsimons et al., 2016; Schouten et al., 2020). The observed dampened circadian oscillations in GC secretion may be part of the mechanisms behind the lack of effect of FLX treatment (Huang and Herbert, 2006). DMI, instead, which has been shown to enhance GR signaling (Pariante et al., 1997), reversed the depression-like phenotype and restored hippocampal neurogenesis and the morphology of newly generated granule neurons (Conti et al., 2017).

### Spontaneous activity and circadian rhythms

Monitoring spontaneous activity (by tracking locomotor activity inside the cage) of group-housed mice in homecage environment provides insight complementary to classical testing (e.g., exploration in open field). In addition, the information is readily translatable to clinical research. The analysis of circadian rhythms in constant 12:12 h light-dark cycle showed slightly increased amplitude in 12 mo-old mice exposed to DEX *in utero*, and a shorter duration of active phase as compared to controls. This can be due to spontaneous activity being restricted to the dark phase only, which may suggest that activity is suppressed outside the dark (active) phase. To further characterize the alterations, we analyzed in detail the patterns of activity around the transitions between light and dark. Animals with intact internal clock regulating activity display anticipatory behavior, which is visualized as gradual increase in activity before the onset of dark phase, or any other events with circadian regularity (e.g., in time-restricted feeding experiments) (Luby et al., 2012). Similarly, spontaneous activity tapers toward the anticipated end of the active phase and continues to low levels for a short time into the beginning of the light (inactive) phase. The mice exposed to DEX *in utero* showed slightly delayed onset, and earlier offset of activity as compared to controls, which effectively restricted their spontaneous activity to the duration of the dark phase. This indicates alterations in circadian entrainment and suggests that the regularity of light-dark cycle is not embedded in the regulation of activity. Instead, the mice exposed to DEX *in utero* merely react to phase change and display limited, if any, prediction of timing of transition between light and dark (Spulber et al., 2015). To address the suspected alterations in photic entrainment, we expanded the testing conditions to include free running (i.e., continuous darkness). In free-running conditions, the internal clock, located in the suprachiasmatic nucleus in the anterior hypothalamus, is the main driver of fluctuations in activity (Inagaki et al., 2007). Interestingly, DEX-exposed mice were undistinguishable from controls in free-running conditions regarding internal circadian period or complexity of activity patterns. Resuming the light-dark cycle after a period of free-running poses the challenge of re-entraining circadian rhythms, and in controls the internal period increased to 24 h after ∼3 cycles and circadian patterns of activity aligned to the light-dark cycle (Spulber et al., 2015). In contrast, DEX-exposed mice re-entrained virtually instantaneously, suggesting that the light-dark cycle was the main driver of circadian fluctuations in spontaneous activity, with minimal contribution from the suprachiasmatic nucleus (Spulber et al., 2015, 2019). This phenotype was detectable already from 1.5 months of age (i.e., the earliest age when it was technically possible to assess circadian rhythms in spontaneous activity), is established around the age of 6 mo, and by the age of 12 mo spontaneous activity appears to follow passively the light-dark cycle (Spulber et al., 2015). These findings suggest a weaker control of the suprachiasmatic nucleus on regulation of spontaneous activity. Therefore, we designed a test to capture the sensitivity to photic re-entrainment, namely the analysis of response to a 6-h advance in onset of dark phase (phaseshift). Advancing the onset of dark phase would allow but not trigger behavioral activation, and the onset of activity changes progressively to match the shifted light-dark cycle, a process regulated by dopaminergic input to the SCN in control mice (Grippo et al., 2017). Starting from about 5 months of age, photic re-entrainment took 3-5 light-dark cycles in controls, while mice expose to DEX *in utero* shift the onset of activity without delay (Spulber et al., 2015; Conti et al., 2017). To assess the coupling between SCN and downstream oscillators, we compared the oscillations in clock gene expression in hippocampus (peripheral oscillator) vs. the SCN (central clock). Photic entrainment of the SCN was found to be intact, while downstream coupling between SCN and the hippocampus was abolished (Spulber et al., 2019). Remarkably, the uncoupling of peripheral oscillators from the SCN was present in males, but not in females exposed to DEX *in utero* (Elberling et al., 2023).

### Depression-like behavior and the response to different antidepressants

We assessed learned helplessness using FST in control and DEX-exposed mice starting from 1.5 mo and observed a significant increase in immobility time in DEX-exposed only at the age of 12 months (roughly equivalent to middle age in humans). Of note, immobility time increased only in male offspring, while female littermates exhibited shorter floating time as compared to controls, consistent with spontaneous hyperactivity (Spulber et al., 2015; Elberling et al., 2023). To reverse depression-like behavior in male mice exposed to DEX were treated with Fluoxetine (FLX, an SSRI-class antidepressant) or Desipramine, a specific noradrenaline reuptake inhibitor (SNRI) class antidepressant) in drinking water for at least 3 weeks before testing. Interestingly, only DMI was effective in reducing immobility time in FST.

There is evidence in the human population that circadian disruption increases the risk for developing depression, and changes in circadian rhythms precede the onset of depression (Edgar and McClung, 2013; Lyall et al., 2018). In our model, the alterations in circadian entrainment in male mice were well-established at the age of 6 mo, but depression-like behavior was not detected. Therefore, we set out to investigate (1) the mechanisms behind the alteration in circadian entrainment; and (2) whether treating the mice with DMI reverses the alterations and prevents the onset of depression-like behavior. The investigation of GR signaling at the age of 6 mo showed downregulated GR expression, as well as lower density of nuclear GR-GR homodimers (active receptors) (Spulber et al., 2019). A decrease in GR-mediated signaling may account for the uncoupling between SCN and hippocampal molecular clocks. At this age, DMI treatment enhanced GR signaling, as shown by upregulated GR expression, and increased density of cytosolic GR-Hsp90 heterodimers (inactive GR) as well as nuclear GR-GR homodimers (active GR) (Spulber et al., 2019). The restoration of coupling between SCN and peripheral oscillators by DMI is illustrated by the coupling of oscillations in clock gene expression between SCN and hippocampus; the photic entrainment of spontaneous activity; and the increased amplitude of oscillations in clock gene expression in skin fibroblasts in culture (Spulber et al., 2019). Remarkably, male mice exposed to DEX *in utero* and treated with DMI at 6 mo did not develop depression-like behavior at 12 mo. Furthermore, the alterations in hippocampal neurogenesis and morphology were considerably reduced by the age of 12 mo (Spulber et al., 2019). One can speculate that enhancing GR signaling and restoring GC-driven circadian entrainment has a protective role against late-onset depression-like behavior in DEX-exposed male mice. Notably, hippocampal neurons express GR, and the hypothalamic secretion of CRH is suppressed by inhibitory projections from the hippocampus (Mastorakos and Ilias, 2003). Taken together, our data indicates that altered GR signaling has an important contribution to the phenotype we observed in male mice exposed to DEX *in utero*.

**FIGURE 3:**
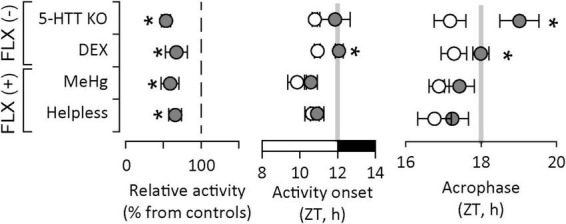
Alterations in circadian patterns of activity in experimental models of depression. Decreased overall activity is a common feature for all models. Altered circadian entrainment, as illustrated by delayed onset of active phase and delayed acrophase (i.e., the time of day when the circadian peak of activity is expected to occur) is found only in experimental models of depression which do not respond to fluoxetine [FLX(-)], an antidepressant drug in the SSRI class. Activity recordings in “helpless” and 5-HTT KO mice courtesy of Dr. Daniela Popa, Institut de Biologie de l’École Normale Supérieure, Paris, France). ZT – circadian (zeitgeber) time; light on between ZT0 and ZT12, light off between ZT12 and ZT24/ZT0. Asterisks indicate significant differences from controls (white symbols or reference dashed line).

We hypothesized there may be a correlation between specific alterations in activity patterns (particularly circadian entrainment) and response to antidepressants. To this end we re-analyzed our data acquired in other experimental models of depression based either on prenatal insults in wildtype mice, or genetically modified animals, in which the effectiveness of different antidepressant classes has already been established. This investigation included (1) wildtype mice exposed to methylmercury (MeHg), an established environmental developmental neurotoxicant (Onishchenko and Ceccatelli, 2010; Onishchenko et al., 2012), in which we have shown that depression-like behavior is reversed by FLX (Onishchenko et al., 2007, 2008; Onishchenko and Ceccatelli, 2010); (2) “helpless” mice, a line selectively bred overexpressing 5-HT1A receptor, in which depression-like behavior is reversed by FLX (El Yacoubi et al., 2003); and (3) serotonin transporter knock-out (5-HTT KO), in which depression-like behavior is reversed by DMI, but not by FLX (Holmes et al., 2002). The analysis of spontaneous activity in the homecage yielded remarkable differences and similarities in alterations across models (Figure 3). First, activity during the active phase is reduced in all models. This is compatible with the decrease in relative amplitude in circadian rhythms associated with depression symptoms (Lyall et al., 2018). When we analyzed circadian entrainment specifically, we found that activity onset was delayed only in experimental models which would not respond to FLX, namely 5-HTT KO and DEX exposure. This suggests that anticipatory behavior, i.e., activation anticipating the onset of dark/active phase, is impaired in these models. Moreover, the acrophase, i.e., the time of day when the circadian peak of activity is expected as predicted by cosinor analysis, was delayed also only in 5-HTT KO and DEX-exposed mice. This suggests that the active phase is delayed as compared to controls and confirms the impairment in anticipatory behavior. In DEX-exposed mice, we have shown that uncoupling between the SCN – where photic entrainment is not altered – and downstream clocks is due to reduced GR signaling. In 5-HTT KO mice, considered to be a reliable SSRI-resistant depression model, the lack of functional 5-HTT increases the availability of 5-HT at synaptic level. In the SCN, 5-HT effectively blocks photic entrainment by inhibiting glutamatergic signaling from the RHT (Reghunandanan and Reghunandanan, 2006; Pontes et al., 2010). Therefore, downstream photic entrainment of spontaneous activity is not possible, leading to initiation of active phase passively following the light-dark cycle instead of anticipating the transitions between light and dark.

In contrast, in depression models which respond to FLX, circadian entrainment of spontaneous activity appears not to be altered. In “helpless” mice, the 5-HT1A autoreceptor upregulation decreases 5-HT availability at synaptic level by inhibiting synaptic release, which explains the depression-like behavior documented in this mouse line. 5-HT signaling in the SCN weakens photic entrainment, and reducing 5-HT availability is not expected to have a significant impact on circadian entrainment. Lastly, in the MeHg-exposure model of depression, there is limited information on mechanisms linked to circadian entrainment. We have shown that mercuration of GR at Cys736 distorts the conformation of the ligand binding site and reduces its activation upon ligand binding (Spulber et al., 2018), which may impact the regulation of HPA axis. This mechanism may be relevant during early stages of development only, since the concentration of MeHg in the brain dropped to control levels within 4 weeks after birth, while persistent changes lasting into adulthood may be accounted for altered BDNF expression due to epigenetic changes (Onishchenko et al., 2008). Conversely, we can speculate that altered BNDF signaling may contribute to the phenotype observed in DEX-exposed mice. Recent reports indicate that maternal leads to epigenetic changes in BNDF promoter region (Braithwaite et al., 2015; Uwaya et al., 2016; Niknazar et al., 2017; Park et al., 2018; Pallarés et al., 2021; Fransquet et al., 2022). However, there is limited data available on BNDF signaling in mice exposed to DEX *in utero*.

### Sex-related differences

Historically neuroscience research has favored a bias against female subjects. While in recent years this trend has decreased, many studies that include both sexes do not consider sex as an experimental variable and this concerns both animal and human research. In addition, sex is rarely considered in research based on *in vitro* cultures of primary cells or immortalized cell lines. Not including sex in the experimental design and analyses may undermine the relevance of studies aiming at clarifying mechanisms of disease. Furthermore, the lack of inclusion of females leads to an underrepresentation of an entire segment of the population, which in turn, can negatively affect our understanding of the impact of specific diseases and the development of new treatment strategies (Mamlouk et al., 2020). The biological underpinnings of sex-related differences in response to prenatal exposure to excess GC include differences between male and female placenta function; differences in reprograming of the HPA axis; and epigenetic changes [reviewed in Carpenter et al. (2017)].

Our model of prenatal exposure to DEX *in utero* revealed strong sexual dimorphism in long-term outcomes of neurodevelopmental insults. In contrast to males, female offspring exposed to DEX displayed spontaneous hyperactivity in a familiar environment (compatible with ADHD-like phenotype). Similarly, female, but not male rat offspring exposed to chronic unpredictable mild stress between GD14 and birth have also been reported to be hyperactive (Possamai-Della et al., 2023). In addition, the ACTH secretion was higher in females as compared to male offspring (Possamai-Della et al., 2023), which is in line with the delayed photic re-entrainment of activity observed in DEX-exposed female offspring (Elberling et al., 2023). Of note, an increase in histone acetylation the hippocampus was reported in female offspring exposed to prenatal stress, but not in males (Possamai-Della et al., 2023). The analysis of impact of phaseshift on the organization of behavior highlighted fundamental differences between males and females. In males, the phaseshift had only minor and reversible effects on general organization of behavior of individual mice. In contrast, phaseshift was followed by widespread and persistent changes in the organization of behavior in females (Elberling et al., 2023). Gene expression analyses have shown that the coupling between SCN and peripheral oscillators is preserved. We further found decreased dopaminergic signaling, which may delay the photic entrainment of the SCN (Grippo et al., 2017; Grippo and Güler, 2019) and account for the overall hyperactivity. Consistent upregulation of Gsk3b in both SCN and hippocampus indicates destabilization of molecular clocks in peripheral oscillators, as previously shown in psychiatric conditions associated with altered clock function (e.g., ADHD, bipolar disorder) (Hirota et al., 2008; Paul et al., 2012; Zhang et al., 2013; Yen et al., 2015). The patterns of behavioral alterations between males and females suggest that the organization of behavior in the homecage is largely independent from photic entrainment. In addition, they highlight sex-related differences in the response to prenatal insults and susceptibility to multifactorial neurodevelopmental disorders.

## Relevance of the findings

Our data indicate that IUGR induced by prenatal exposure to DEX is an experimental model of late-onset depression characterized by altered circadian entrainment of activity, and selective response to antidepressants (no response to FLX, positive response to DMI). The association between circadian entrainment and response to antidepressant treatment in animal models of depression can be explained as outcomes of specific mechanisms, and it has been verified in specific cases. In the *in utero* exposure to DEX model, we found evidence of effective antidepressant treatment restoring the alterations in circadian entrainment (Spulber et al., 2019), but there is virtually no data available in other models. However, circadian entrainment is an endpoint which can be evaluated in patients using non-invasive, affordable technologies for monitoring activity, such as wrist actigraphy. A large biobank study has shown that decreased relative amplitude (RA) of circadian rhythms of activity correlates with increased susceptibility to mood disorders and poorer subjective wellbeing (Lyall et al., 2018). RA measures how distinct the levels of activity are during the least active interval from the most active interval, regardless of the time-of-day when they are detected (i.e., no assumption regarding intrinsic circadian periodicity). Therefore, decreased RA (due to either higher activity at night; lower diurnal activity; or a combination of both) reflects less distinct circadian modulation of activity, which is compatible with weaker circadian entrainment. We have developed a pipeline for detailed analysis of individual patterns of activity in MDD patients focusing on circadian entrainment and within-day variability of activity. Initial analyses identified correlations between patterns of activity and symptom severity (Spulber et al., 2022). We further explored the possibility to model the response to specific antidepressant interventions using individual patterns of activity during depressive episode but before treatment using Bayesian model averaging on independently trained multivariate linear regression models. These analyses revealed substantially different subsets of features to be most relevant for specific interventions (Spulber et al., 2023, 2025). These results highlight the possibility to introduce actigraphy recordings as objective measurements to assist mental healthcare. The predicted increase in remission rate using informed assignment to treatment was estimated to 35% (Spulber et al., 2025), which is similar to evidence-based care and algorithm-guided therapy (Xiao et al., 2021). Given the limited information available, prospective clinical trials are required for validation of models before introduction in clinical practice.

### Perspectives

It is relevant to point out that experimental models can resolve mechanisms which are sufficient to yield symptoms listed among diagnostic criteria for MDD. For instance, genetically engineered models targeting genes associated with depression (e.g., related to 5-HT signaling, or clock genes), or models where the HPA axis functions is reprogrammed by prenatal interventions (e.g., prenatal stress, or exposure to synthetic GC) lead to depression-like behavior in mice. These models provide insight into phenotypes associated with the response to different antidepressant interventions. Since the alterations appear specific to an underlying mechanism, they may indicate which antidepressant treatments are effective and which are expected to not be effective. From a translational perspective, these results suggest that alterations in patterns of circadian spontaneous activity may predict the response to therapy in depressed patients. There are no objective measures or biomarkers to predict the response to specific antidepressant treatment, and to date effective therapies are identified by trial and error. A recent extensive review analyzing individual trajectories in response to treatment shows that the patterns of response to treatment are consistent across drugs (Stone et al., 2022). While successful antidepressant response can be predicted by clinical features, it is not possible to predict the specific antidepressant to which a patient will respond before treatment initiation. Therefore, it is meaningful to search for predictors of response to specific antidepressant interventions (Stone et al., 2022). Activity patterns in patients can be monitored using wrist actigraphy. Our collaborative clinical investigations provided evidence for the prognostic value of circadian patterns of activity for predicting the response to antidepressant treatments (Spulber et al., 2023, 2025). Despite limitations due to population size, the use of transparent machine learning algorithms which account for uncertainty around data-generating model and support biological interpretations make possible the translation of model parameters into clinically relevant information.
